# Supplementary material for: Mutational bias in spermatogonia impacts the anatomy of regulatory sites in the human genome
Source: Genome Res. 2021 Nov;31(11):1994–2007. doi: 10.1101/gr.275407.121 (PMC8559717; doi:10.1101/gr.275407.121)
Supplement: Supplemental Material [file supp_31_11_1994__DC1.html]

Mutational bias in spermatogonia impacts the anatomy of regulatory sites in the human genome — Supplemental Material 

# Mutational bias in spermatogonia impacts the anatomy of regulatory sites in the human genome

## Supplemental Material

- Supplemental\_Material.docx
- Supplemental\_Tables\_S1\_to\_S12.xlsx
- Supplemental\_Data.zip
