# Supplementary material for: Mutational bias in spermatogonia impacts the anatomy of regulatory sites in the human genome
Source: Genome Res. 2021 Nov;31(11):1994–2007. doi: 10.1101/gr.275407.121 (PMC8559717; doi:10.1101/gr.275407.121)
Supplement: Supplemental Material [file supp_gr.275407.121_Supplemental_Material.docx]

**Mutational bias in spermatogonia impacts the anatomy of regulatory sites in the human genome**

Vera B. Kaiser^1^, Lana Talmane^1^, Yatendra Kumar^1^, Fiona Semple^1^, Marie MacLennan^1^, Deciphering Developmental Disorders Study^1,2^, David R. FitzPatrick^1^, Martin S. Taylor^1*^, Colin A. Semple^1*^

^1^MRC Human Genetics Unit, MRC Institute of Genetics and Cancer, The University of Edinburgh, Western General Hospital, Crewe Road South, Edinburgh EH4 2XU, UK

^2^The Wellcome Trust Sanger Institute, Wellcome Trust Genome Campus, Hinxton, Cambridge, CB10 1SA, UK

*Equal contribution

Corresponding author: Vera B Kaiser vera.kaiser@ed.ac.uk

**Supplemental Material**

Supplemental Methods: p. 2-4

Supplemental Figures: p. 5-14

**Supplemental Methods**

**Processing of raw sequencing reads**

ATAC-seq raw reads were trimmed to remove any retained adaptor sequences using cutadapt (Martin 2011) with parameters *-n3 --format=fastq --overlap=3* *-g GAGATGTGTATAAGAGACAG -g CAGATGTGTATAAGAGACAG –a CTGTCTCTTATACACATCTG -a CTGTCTCTTATACACATCTC.*Reads were aligned to the GRCh38/hg38 genome assembly with Bowtie 2 (Langmead and Salzberg 2012) in paired-end mode, limiting the insert size to 4 kb. Any reads with quality score < 30 were discarded. Paired end reads were converted to fragments using BEDTools’ bedtools bamtobed -bedpe, followed by extraction of the most 5’ and 3’ coordinates of each pair. PCR duplicates were removed by retaining only one instance of a fragment with identical coordinates within a sample. Fragments overlapping with the regions previously blacklisted as mitochondrial homologs (Buenrostro, Giresi et al. 2013) were discarded. Peaks were identified from short fragments of <= 100 bp (Supplemental Fig. S1), thought to arise due to transposition events around transcription factor binding sites – and distinct from fragments spanning the larger nucleosomes (characterized by a ~200 bp periodicity) (Buenrostro, Giresi et al. 2013). Peaks were called from short fragments using macs2 callpeak (Zhang, Liu et al. 2008) with the following parameters: -B -q 0.01 -f BAMPE --nomodel --nolambda --keep-dup auto --call-summits. The clustering of ATAC-seq peaks near transcription start sites and promoters was assessed using the ChIPseeker R package (Yu, Wang et al. 2015).

**Comparisons between ATAC-seq datasets**

BedGraph files (from the MACS2 output), describing the fragment pileup, were converted to bigWig format using bedGraphToBigWig and uploaded to the Galaxy server at https://usegalaxy.eu/(Afgan, Baker et al. 2018). DeepTools2’s multiBigwigSummary (with default parameters) and plotCorrelation (with parameters –skipZeros –removeOutliers) (Ramirez, Ryan et al. 2016) were used to create a heatmap of ATAC-seq signals in the different tissues, including ENCODE and spermatogonial datasets. The KIT+ and SSEA4+ spermatogonial datasets of Guo et al. (2017) and Guo et al. (2018) were further used to perform footprinting and motif matching analyses (Li, Schulz et al. 2019) as described above for the FGFR3-positive cells. Peaks and motif sites that are accessible in the developing brain but not in spermatogonia were identified using BEDTools’ bedtools intersect (Quinlan and Hall 2010).

**Circular Permutations**

At each iteration of circular permutations (Gel, Diez-Villanueva et al. 2016), genomic positions are shifted by the same randomly generated distance on conceptually "circularized" chromosomes, in effect “spinning” the position of features on each chromosome, while excluding masked regions (i.e. unmappable, repetitive and low-complexity segments). The statistical output is a *Z*-score, which is defined as the distance between the expected number of overlaps (the distribution over 10,000 permutations) and the observed one, measured in terms of standard deviations. Shifted *Z*-score analysis can further indicate whether a given overlap pattern is caused by the precise locations of two feature sets or by broader, regional effects. One set of features is shifted in either direction from their original positions by a number of bases, so that the numbers of overlaps and the degree to which the *Z*-score changes in response to the shift can be tested. A sharp peak of shifted *Z*-scores around the zero-coordinate indicates a precise overlap between features, whereas a flat profile may indicate overlaps attributable to regional effects, as is seen for features that tend to co-occur in regions with similar base composition or gene density.

**Simple permutations**

Spermatogonial binding sites were randomly permuted, using BEDTools’ bedtools shuffle command (Quinlan and Hall 2010), with the parameter “-noOverlapping” and excluding assembly gaps with “-excl hg38.gap.bed” (downloaded from https://genome.ucsc.edu/cgi-bin/hgTables). In each of 10,000 simulations, we assessed the overlap of the permuted binding sites with short insertions and deletion breakpoints, respectively. This resulted in an expected distribution of overlap, given a random positioning of binding sites across the genome. This distribution was compared to the observed number of overlaps (using BEDTools’ bedtools intersect), and the p-value was defined as the percentage of simulated overlaps that was larger than the observed overlap. This permutation framework does not take into account the spacing and clustered nature of binding sites, and does not allow for an assessment of the precision of overlap between features. In this study, we favor the more conservative measures of significance provided by the circular permutation strategy.

**Supplemental Figures**


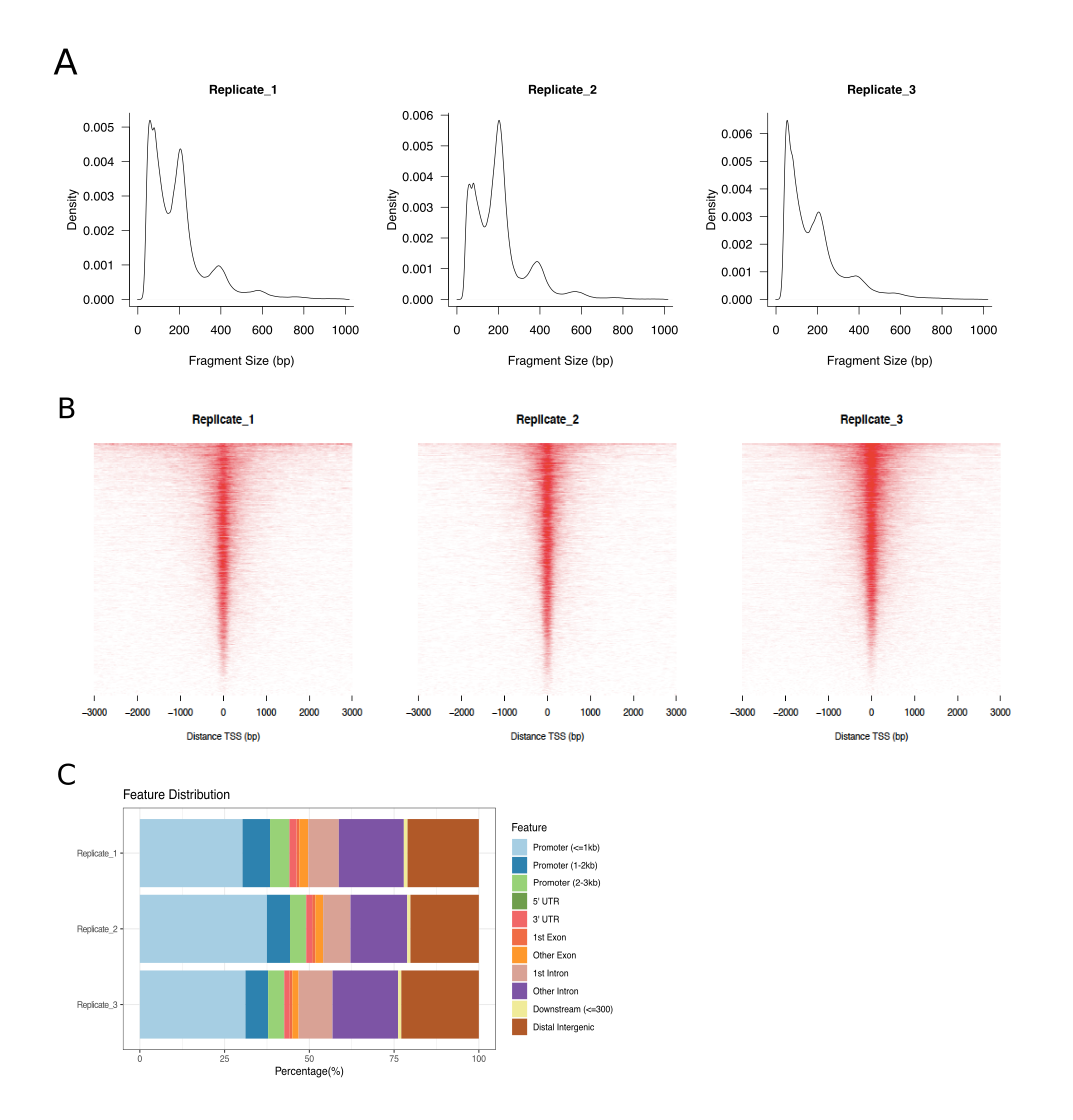


**Figure S1: Summary of spermatogonial ATAC-seq data.** (A) Fragment length distribution, showing the ~200bp periodicity around nucleosome-free regions; fragments <=100bp in length were chosen for downstream analysis and peak calling. (B) Heatmap of ATAC-seq peak density around transcription start sites (TSSs). (C) Genomic feature annotation of ATAC-seq peaks. (B) and (C) were produced using ChIPseeker, using the UCSC hg38 genome annotation.

**Figure S2: Clustered heatmap of ATAC-seq datasets.** Colours indicate the correlation coefficients between the genome-wide ATAC-seq signals in SSEA4+ spermatogonial stem cells (SSC) (Guo, Grow et al. 2017); KIT+ SSC (Guo, Grow et al. 2018); FGFR3+ spermatogonia (this dataset); ENCODE tissues (The ENCODE Project Consortium 2012, Davis, Hitz et al. 2018); ESC cells (Guo, Grow et al. 2017); the germinal zone and cortical plate of the developing brain (de la Torre-Ubieta, Stein et al. 2018).

**Figure S3: The enrichment of short variants and SV breakpoints at spermatogonial binding sites, using positive and negative controls.** The Y axis shows the ratio of observed over expected variant counts at accessible sites, based on 10,000 circular permutations. Mutation categories with significant enrichment or depletion are indicated by asterisks (* = *p* < 0.05; ** = *p* < 0.01 *** = *p* < 0.001). The type of variant tested and the total number of observed variants overlapping TFBSs are indicated below each bar. (A) Sites accessible in Replicate 3 (FGFR3+ spermatogonial cells). (B) Sites that are accessible in the germinal zone and/or cortical plate (de la Torre-Ubieta et al. 2018), but not accessible in any of the spermatogonial samples (FGFR3-, SSEA4- or KIT-marked spermatogonia).


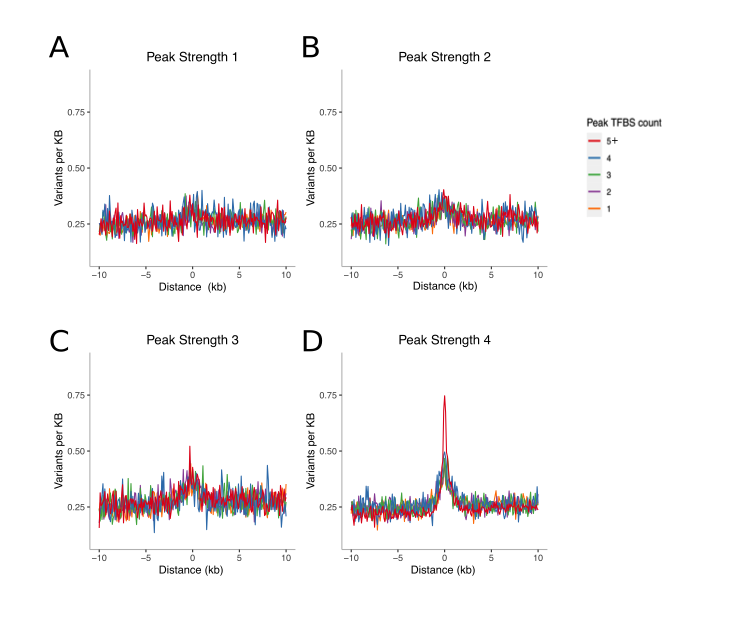


**Figure S4: Short (5-20 bp) insertion rates, stratified by peak strength and the number of TFBSs within peaks.** (A-D) Spermatogonial ATAC-seq peaks were divided into four equal sized groups, based on their quartile of peak score (after removal of outliers, i.e. the top 1% of peak scores): Peak Strength 1, 2, 3 and 4. Next, the peaks were classified by the number of putative binding sites that they contained, ranging from 1 TFBS to “5 or more” TFBSs. The X axis shows the distance from the centre of the ATAC-seq peak, and the Y axis shows the average rate of singleton insertions ( 5-20bp ) in the gnomAD dataset, for each category of ATAC-seq peaks.

**Figure S5:** **Short insertions (5-20 bp) and deletion breakpoints are correlated.** (A) Rates of singleton gnomAD insertions and DDD deletion breakpoints, for 72 motif families with at least 3,000 predicted binding sites in spermatogonia; each data point represents one motif family. PRDM9 is highlighted in red. (B, C) Circular permutation *Z*-scores (left axis, black dots) and the enrichment of variants for each motif family (the ratio of observed over expected numbers of variants; right axis, grey stars). PRDM9 is highlighted in red and pink, respectively. Motifs are ranked by ranked by *Z*-score (Motif Index).

**Figure S6: SV deletion breakpoints often precisely overlap InDels (5-20bp).** The X-axis is centered around deletion breakpoints of the gnomAD structural variant dataset. Each data point indicates the aggregate number of gnomAD short variants (SNPs and InDels, respectively) observed at a given distance from the breakpoint, at basepair resolution. **Red**: singleton short variants *versus* singleton deletion breakpoints. All singleton short variants were down-sampled to a total of 650,000 variants each, making the Y axes comparable. **Blue**: High frequency short insertions (5-20bp) (*p* >= 5%) *versus* high frequency deletion breakpoints (*p* >= 5%).

­­­

**Figure S7**: **Rate of singleton InDels and snps in the gnomAD dataset near active spermatogonial motif sites.** Insertions, Deletions and SNPs were down-sampled to a total of 650,000 variants each, making the Y axes comparable; individual bins are 5 bp in size. The ten motifs shown had the highest overall number of observed insertions of 5-20 bp (Supplementary Table 4). Only regions around TFBSs with >=95% unique mappability (umap24 scores) were included.

**Figure S8: Insertions often cause duplications of the binding motif.** JASPAR database sequence motifs in the footprints of spermatogonial ATAC-seq peaks (left) and the motifs identified by meme in the singleton insertions (5-20bp) at these sites (right). Shown are the ten motif families with the overall highest number of insertions. A maximum of three JASPAR motifs per motif family are shown on the left.

**Figure S9: Random Forest analysis of insertion (5-20 bp) and deletion breakpoint rates.** The importance of predictor variables, measured as the % increase in mean square error when the variable is removed from the model (%IncMSE), is plotted for four different random forest regression models. (A, B) modelling mutation rates genome-wide (in 5 kb-wide bins). (C, D) modelling mutation rates in 5 kb-wide bins which also overlap brain active enhancers as defined in the main text.

A B

**Figure S10: PRDM9 motifs within ATAC-seq footprints are enriched at testis-derived ssDNA sites.** (A) The frequency of PRDM9-binding sites, centred around ssDNA sites. (B) The ssDNA peak strength *versus* the distance to the nearest PRDM9-binding site. ssDNA data came from Pratto, Brick et al. (2014) and PRDM9-binding sites from the spermatogonial footprinting analysis.

**References**

Afgan, E., D. Baker, B. Batut, M. van den Beek, D. Bouvier, M. Cech, J. Chilton, D. Clements, N. Coraor, B. A. Gruning, A. Guerler, J. Hillman-Jackson, S. Hiltemann, V. Jalili, H. Rasche, N. Soranzo, J. Goecks, J. Taylor, A. Nekrutenko and D. Blankenberg (2018). "The Galaxy platform for accessible, reproducible and collaborative biomedical analyses: 2018 update." Nucleic Acids Res **46**(W1): W537-W544.

Buenrostro, J. D., P. G. Giresi, L. C. Zaba, H. Y. Chang and W. J. Greenleaf (2013). "Transposition of native chromatin for fast and sensitive epigenomic profiling of open chromatin, DNA-binding proteins and nucleosome position." Nat Methods **10**(12): 1213-1218.

Davis, C. A., B. C. Hitz, C. A. Sloan, E. T. Chan, J. M. Davidson, I. Gabdank, J. A. Hilton, K. Jain, U. K. Baymuradov, A. K. Narayanan, K. C. Onate, K. Graham, S. R. Miyasato, T. R. Dreszer, J. S. Strattan, O. Jolanki, F. Y. Tanaka and J. M. Cherry (2018). "The Encyclopedia of DNA elements (ENCODE): data portal update." Nucleic Acids Res **46**(D1): D794-D801.

de la Torre-Ubieta, L., J. L. Stein, H. Won, C. K. Opland, D. Liang, D. Lu and D. H. Geschwind (2018). "The Dynamic Landscape of Open Chromatin during Human Cortical Neurogenesis." Cell **172**(1-2): 289-304 e218.

Gel, B., A. Diez-Villanueva, E. Serra, M. Buschbeck, M. A. Peinado and R. Malinverni (2016). "regioneR: an R/Bioconductor package for the association analysis of genomic regions based on permutation tests." Bioinformatics **32**(2): 289-291.

Guo, J., E. J. Grow, H. Mlcochova, G. J. Maher, C. Lindskog, X. Nie, Y. Guo, Y. Takei, J. Yun, L. Cai, R. Kim, D. T. Carrell, A. Goriely, J. M. Hotaling and B. R. Cairns (2018). "The adult human testis transcriptional cell atlas." Cell Res **28**(12): 1141-1157.

Guo, J., E. J. Grow, C. Yi, H. Mlcochova, G. J. Maher, C. Lindskog, P. J. Murphy, C. L. Wike, D. T. Carrell, A. Goriely, J. M. Hotaling and B. R. Cairns (2017). "Chromatin and Single-Cell RNA-Seq Profiling Reveal Dynamic Signaling and Metabolic Transitions during Human Spermatogonial Stem Cell Development." Cell Stem Cell **21**(4): 533-546 e536.

Langmead, B. and S. L. Salzberg (2012). "Fast gapped-read alignment with Bowtie 2." Nat Methods **9**(4): 357-359.

Li, Z. J., M. H. Schulz, T. Look, M. Begemann, M. Zenke and I. G. Costa (2019). "Identification of transcription factor binding sites using ATAC-seq." Genome Biology **20**.

Martin, M. (2011). "Cutadapt Removes Adapter Sequences From High-Throughput Sequencing Reads." EMBnet.journal **17**(1): 10-12.

Pratto, F., K. Brick, P. Khil, F. Smagulova, G. V. Petukhova and R. D. Camerini-Otero (2014). "DNA recombination. Recombination initiation maps of individual human genomes." Science **346**(6211): 1256442.

Quinlan, A. R. and I. M. Hall (2010). "BEDTools: a flexible suite of utilities for comparing genomic features." Bioinformatics **26**(6): 841-842.

Ramirez, F., D. P. Ryan, B. Gruning, V. Bhardwaj, F. Kilpert, A. S. Richter, S. Heyne, F. Dundar and T. Manke (2016). "deepTools2: a next generation web server for deep-sequencing data analysis." Nucleic Acids Res **44**(W1): W160-165.

The ENCODE Project Consortium (2012). "An integrated encyclopedia of DNA elements in the human genome." Nature **489**(7414): 57-74.

Yu, G., L. G. Wang and Q. Y. He (2015). "ChIPseeker: an R/Bioconductor package for ChIP peak annotation, comparison and visualization." Bioinformatics **31**(14): 2382-2383.

Zhang, Y., T. Liu, C. A. Meyer, J. Eeckhoute, D. S. Johnson, B. E. Bernstein, C. Nussbaum, R. M. Myers, M. Brown, W. Li and X. S. Liu (2008). "Model-based Analysis of ChIP-Seq (MACS)." Genome Biology **9**(9).
